# Supplementary material for: Larix species range dynamics in Siberia since the Last Glacial captured from sedimentary ancient DNA
Source: Commun Biol. 2022 Jun 9;5:570. doi: 10.1038/s42003-022-03455-0 (PMC9184489; doi:10.1038/s42003-022-03455-0)
Supplement: Supplementary file 2 — Supplementary Information [file 42003_2022_3455_MOESM2_ESM.pdf]

Supplemental Information

Supplementary Methods

Hybridization capture targeting the chloroplast genome and nuclear genes of *Larix*

Sample material and chronostratigraphy

Sample material was obtained from 8 lake sediment cores from across Siberia, for location and published age model see Supplementary Data 4.

Radiocarbon dating and age modelling of sediment record PG2361, Lake Satagay

We used Bacon in R and the IntCal20 calibration curve to model the age-depth relationship based on 11 radiocarbon dates from the MICADAS laboratory (Bremerhaven, Supplementary Data 5). Two bulk samples 5281.1.1 and 5193.1.1 from 0.5 and 341.75 cm core depth, respectively, were identified as outliers because they contained too high amounts of old carbon compared to a linear relationship with adjacent samples. We used linear regression of C^{14} ages (n=10, excluding the bottom sample 5188.1.1) to find the intersection with the age axis at 0 cm depth and used this value as an old carbon reservoir effect in the lake system (delta.R=1955.2 yrs). The 0.5 cm bulk sample supports the assumption of an old carbon effect in the lake, although it is ca. 570 yrs too old compared to the inferred reservoir value. We used the maximum dating error from the C^{14} dataset as the standard deviation for the reservoir estimate (delta.STD=52 yrs) applied to 11 samples. A dated water plant from a surface sample revealed a roughly modern age (48 +-18 C^{14} yrs at 0-1 cm core depth). Outliers were not used for age-depth modelling but are indicated by red crosses (Fig. S1).

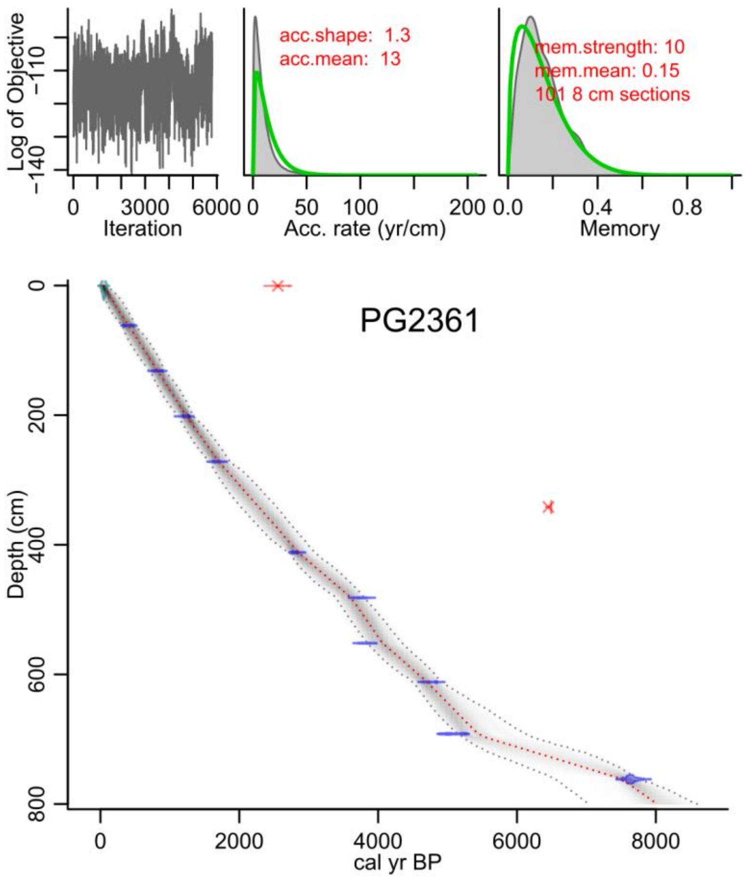

Fig. S1: Age-depth model of PG2361 (Lake Satagay) performed using R package 'Bacon'[1]

Data analysis

Demultipelxed and adapter-trimmed fastqc files as obtained from the sequencing company were quality checked using FASTQC[2] before and after deduplication with CLUMPIFY[3] (v.38.87) and trimming with FASTP[4] (v.0.20.1) with the parameters --merge, --length_required=30, --overlap_len_require=5, --correction, --low_complexity_filter, --cut_front, --cut_tail, --cut_window_size=4, and --cut_mean_quality=10.

Analysis of chloroplast enrichment

DNA sequence reads were classified using KRAKEN2[5] (version 2.1.1) with a confidence threshold of 0.8 against a chloroplast database (RefSeq plastid release of NCBI[6]) downloaded in July 2021. The database was build using kraken2 standard parameters (kmer length of 35 bp and minimizer length of 31 bp). Reads classified to *Larix* at genus or species level were extracted using KRAKENTOOLS extract_kraken_reads.py[7] (v. 0.1). *Larix*-classified reads were aligned against an *L. gmelinii* chloroplast reference genome (NCBI GenBank accession number MK468637) using BWA ALN[8] (v. 0.7.17) with the parameters -l 1024 -o 2 -n 0.01 as recommended for ancient DNA read mapping by Oliva et al.[9]. Further processing of the alignment files such as conversion, sorting and indexing was done using SAMTOOLS[10] (v. 1.11). Duplicates were removed a second time, as deduplication is more efficient in aligned

reads. Deduplication was done using PICARD MARKDUPLICATES[11] (v.2.24.1) for merged and unpaired reads and SAMTOOLS MARKDUP[10] for unmerged reads with default parameters. Ancient damage patterns were assessed and quality scores of likely damaged positions rescaled using MAPDAMAGE2[12] (v. 2.2.1). Alignments produced with overlapping merged reads, unmerged reads and unpaired reads were merged to one bam file using SAMTOOLS MERGE[10].

Variations were called for all samples conjointly using FREEBAYES[13] (v. 1.3.2) with the options --pooled-continuous and --min-base-quality 10. The produced vcf file was processed using PLINK[14] (v.1.90b4) to filter samples with over 90% missing sites and VCFTOOLS[15] (v. 0.1.16) to filter sites for maximal 70% of missing data and a minor allele frequency greater than 1%.

For a comparison of the variations in the samples with known references, all available chloroplast genomes from the Siberian boreal larch species western range *L. gmelinii* (11 species), eastern range *L. gmelinii* (or *L. cajanderi* (7 spec.)) and *L. sibirica* (1 spec.) were downloaded (NCBI GenBank accession numbers: MK468630.1-MK468636.1 and MK468638.1-MK468648.1, NC_036811.1; number of available genomes given in parentheses). All genomes were aligned against the same *L. gmelinii* reference genome as used for the sample MK468637 using BWA MEM[8]. Variations were called conjointly using FREEBAYES[13] with parameter settings --min-alternate-count, 1 --min-alternate-fraction 0, --haplotype-length 0, and --pooled-continuous.

Variations of samples and references were compared and plotted using R[16] and the packages tidyverse[17], readxl[18], cowplot[19] and ggh4x[20] with colors based on Okabe & Ito[21]. Maps were plotted in R using the packages ggmap[22], rgdal[23], sp[24], broom[25] and scatterpie[26].

Analysis of nuclear bait set enrichment

Analysis was conducted as described above. Differences in the analysis are: as database for classification, the plant database of RefSeq was used and 4 genomes of sequenced Pinaceae added using KRAKEN2 (*Picea abies, P. glauca, Pinus taeda,* NCBI GenBank accession numbers GCA_900067695.1, GCA_000411955.6 and GCA_000404065.3). Reads classified to genus *Larix* using KRAKEN2 with a confidence threshold 0.8 were mapped against the set of nuclear genes (see Supplementary Data 2) with the same tools and parameters as described for the analysis of chloroplast reads. As very few reads only mapped to the nuclear genes target probe set, unmapped *Larix*-classified reads were closely inspected using *de-novo* assembly and BLAST in Geneious[27], which revealed a high content of repetitive DNA, in particular of the most abundant satellite repeat of *Larix.* This satellite repeat was first described by Hizume et al.[28] and more thoroughly described by Heitkam et al.[29] in European larch (*L. decidua)* and Japanese larch (*L. kaempferi).* To check whether or not the consensus sequence (most abundant version of the satellite repeat sequence in the genome) differs between the *Larix* species *L. gmelinii,* and *L. sibirica,* a comparative repeat analysis was undertaken (see description in next section). As the consensus sequence in the three species differed only at one position (*L. sibirica* carries at position 96 a cytosine instead of an adenosine), the alignment of *Larix*-classified reads was repeated with the published consensus sequence of EulaSat1[29]. To get reads mapping at the

end of one repeat sequence (in the genome, satellite sequences are arranged in large arrays of repeated monomers), a triplet of the monomer was used as a reference for the alignment.

Enrichment of the satellite repeat

To test whether the repeat sequence became enriched by the capture approach and by which set of baits, we compared three datasets produced from Lake CH12 samples: 1) the un-enriched shotgun dataset (ENA project number PRJEB35838, sample accession numbers SAMEA6430888-91), 2) the target-enriched capture dataset using the complete chloroplast genome as a hybridization probe set (ENA project number PRJEB35838, sample accession numbers SAMEA6430894-97) (datasets 1 and 2 published by Schulte et al.[30]), and 3) the samples of Lake CH12 enriched for the set of nuclear genes produced in this study. Reads were quality trimmed and merged as described above and aligned to the triplet of the EulaSat1 consensus sequence as described above. Percentages of mapped reads to the repeat of the total quality trimmed reads were calculated.

Comparative repeat analysis of *L. gmelinii* and *L. sibirica*

To estimate the amount of repetitive DNA and to quantify EulaSat1 in the three *Larix* genomes, read clustering was performed using the RepeatExplorer Pipeline[31] with standard parameters using paired-end reads of publicly available *Larix* accessions: SRR8555411 (*L. sibirica*), SRR9610223 (*L. gmelinii,* eastern range), and SRR9610240 (*L. gmelinii,* western range). Proportions of sequence families that constitute more than 0.01% of the genome, including long terminal repeat (LTR) retrotransposons, DNA transposons, ribosomal DNA, Long and Short Interspersed Nuclear Elements (LINES/SINES) and satellite repeats, are displayed in Fig. S2. Initial quality filtering of the reads was done using TRIMMOMATIC[32], to provide 1 Mio reads per species with a consistent length of 93 nucleotides (LEADING:5 TRAILING:30 CROP:93 MINLEN:93) for the analyses. Read quality was checked using FastQC[2]. Bowtie2[33] was applied to identify and subsequently remove reads representing plastid DNA from the sequence data by mapping them to a database containing chloroplast DNA of the three individuals (NC_036811.1, MK468640.1, MK468648.1).

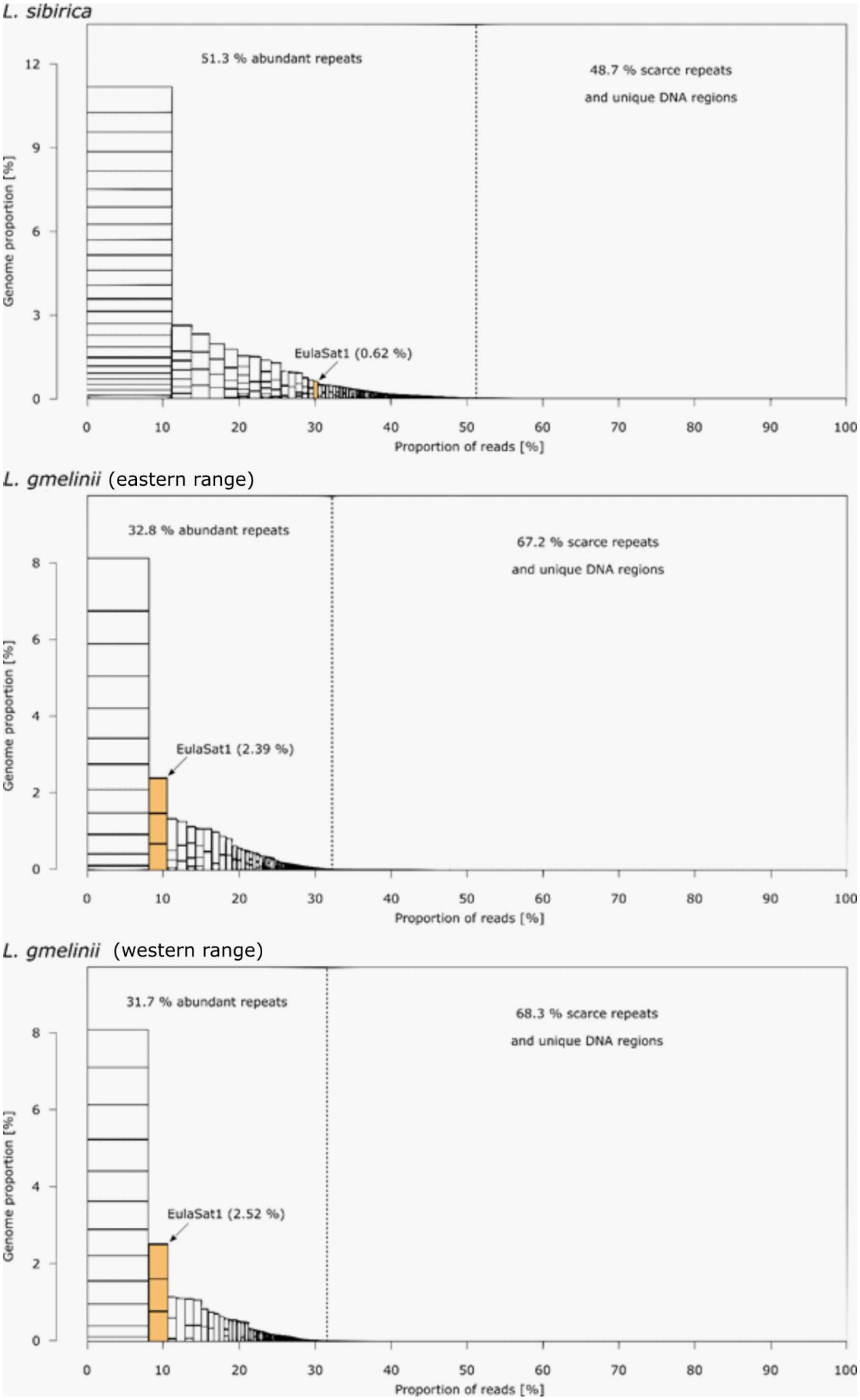

Fig. S2: Quantification of the most abundant repetitive sequences from *Larix sibirica* and *L. gmelinii* (western and eastern range) by genome-wide read clustering. Stacked bars

represent repetitive DNA of the same sequence family, with the X- and Y-axes being a measure of abundance by read proportion. Repeats were considered as either abundant or scarce if their genome proportion exceeds or falls below 0.01% genome proportion, respectively. The read cluster corresponding to EulaSat1 is highlighted in all graphs.

Metabarcoding approach

Sampling, DNA extraction, PCR

To compare results from the hybridization capture approach with the metabarcoding approach, both published and newly produced data were used. Published datasets include Lake CH12[34] and Lake Bolshoye Shchuchye[35]. For lakes Billyakh, Kyutyunda, Lama, Malaya Chabyda and Satagay, new samples were processed (for sample list see Supplementary Data 8). Core sampling and DNA extraction were done as described for the hybridization capture approach. PCR reactions were performed in three independent replicates using the trnL-g and trnL-h primers[36] modified to carry unique 8 bp tags on the 5' prime end preceded by NNN to improve cluster generation[37,38]. PCR set-up, conditions and sequencing were performed as described in Zimmermann et al.[39].

Data analysis of metabarcoding

Published and new metabarcoding datasets were analysed using OBITOOLS[40] (version 3.0.0b38). First, overlapping paired reads were merged using the command *alginpairedend*, then unmerged reads were removed with *grep -a mode:alignment.* The command *ngsfilter* was used to assign each sequence to its corresponding sample according to the tag combination. Consequently, reads were de-replicated with the command *unique* and cleaned from PCR and sequencing errors using *clean -r 0.05* (-r defines the maximum ratio allowed between the counts of sequence variants). Reads were assigned to a taxonomic level using *ecotag.* For taxonomic assignment, two databases were used: 1) a database based on the curated arctic and boreal vascular plant and bryophyte reference database published by Sønstebø et al.[41], Willerslev et al.[42] and Soininen et al.[43] and 2) a database based on the EMBL Nucleotide Database standard sequence release 143[44] (ftp://ftp.ebi.ac.uk/pub/databases/ena/sequence/release/). Databases were produced in OBITOOLS using the *ecopcr* command. In R[16], assignments of both databases were joined, preferentially using the scientific name assigned by the arctic and boreal vascular plant and bryophyte reference database, where the identity was higher or equal to the identity of the EMBL database. Subsequently, reads were filtered for a best identity higher than 0.97, counts for the PCR replicates were summed up and the percentage of reads assigned to *Larix* in the samples calculated.

Pollen

In addition to the Kyutyunda pollen record of core PG2022[45], nine samples of the parallel core PG2023 were selected for pollen analysis (Supplementary Data 12). Standard HF techniques were used for sample preparation[46]. *Lycopodium* marker spores were added to each sample to calculate total spore and pollen concentrations[47]. Water-free glycerol was used for sample storage and preparation of the microscopic slides. Pollen were analyzed using a 400x magnification and identified with the help of published pollen atlases[48–52]. Non-pollen-palynomorphs were identified when possible according to van Geel[53]. At least 250-300 pollen grains were counted in each sample. The relative frequencies of pollen taxa were calculated from the sum of the terrestrial pollen taxa. The percentages of fungal spores are based on the sum of the pollen and fungal spores, and the percentages of algae are based on the sum of pollen and algae. TGView software[54] (v. 1.7.16) was used for the calculation of percentages.

Additional Results & Discussions

Sequencing results

Three datasets were used in the study: 1) the hybridization capture dataset targeting both the chloroplast and a set of nuclear genes of *Larix* in 64 samples and 19 negative controls from seven lake sediment cores, 2) the published hybridization capture data targeting the *Larix* chloroplast in four core samples of Lake CH12 (ENA project number PRJEB35838, sample accession SAMEA6430894-99), and 3) a dataset on the same CH12 core samples targeting the same set of nuclear genes as above. The three datasets comprised in total 1.15 billion paired reads: 946, 201, and 2 million (M) reads for the three datasets, respectively, of which 323, 54, and 1.5 M reads (380 M reads in total) remained after quality filtering and deduplication.

Enrichment of satellite repeat by nuclear gene bait set

To test whether the repeat sequence became enriched by the capture approach and by which set of baits, we compared three datasets produced from the same samples of Lake CH12: 1) the shotgun and 2) target enriched capture datasets produced in the study of Schulte et al. (2020) using the complete chloroplast genome as a bait set and 3) a hybridization capture approach on the same sample libraries using only the nuclear gene bait set. The comparative analysis of the percentage of mapped reads against the sequence of EulaSat1 showed slight enrichment by the chloroplast bait set and a pronounced enrichment by the nuclear gene bait set (Fig. S3). This implies that the enrichment of off-target reads is linked either to the production or sequence composition of hybridization probes, and not a general by-product.

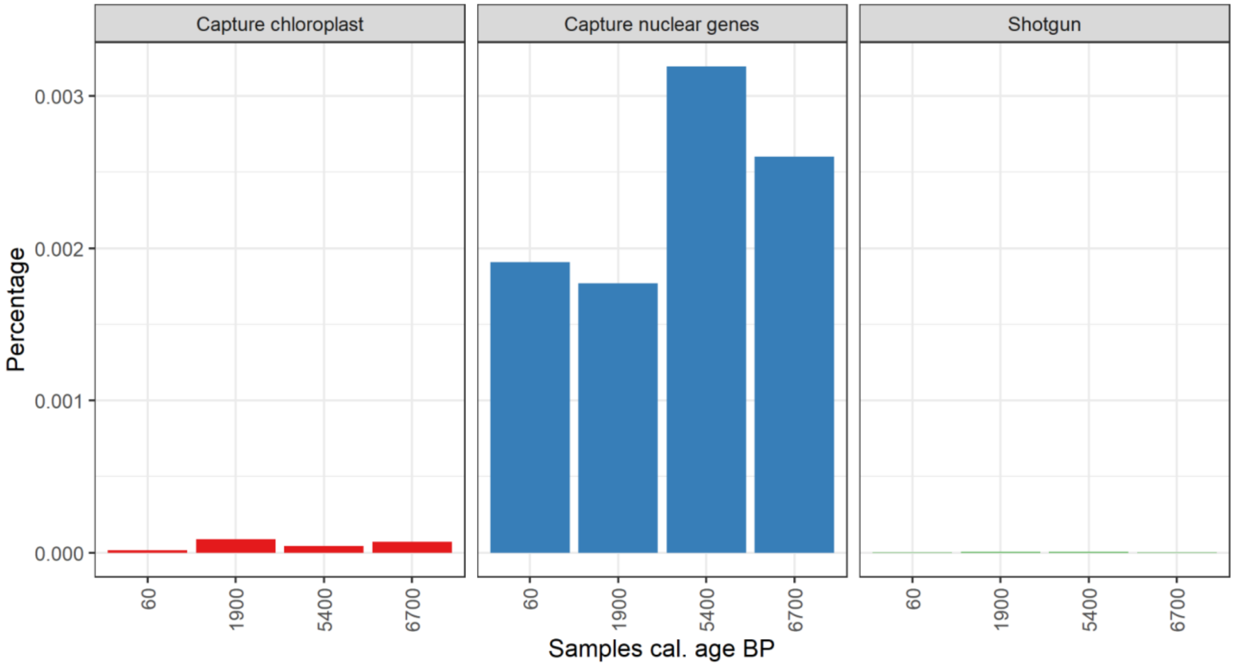

Fig. S3 Percentage of unclassified reads mapped against the EulaSat1 repeat sequence Percentages are displayed for three datasets: 1) capture enrichment using the *Larix* chloroplast genome, 2) capture enrichment using the nuclear bait gene set, and 3) shotgun sequencing. For all sequencing datasets the same libraries were used.

Nucleotide variation in EulaSat1 alignment

The nucleotide variation in the alignment of reads against the consensus sequence of the EulaSat1 repeat shows strong homogeneity (Fig. S4).

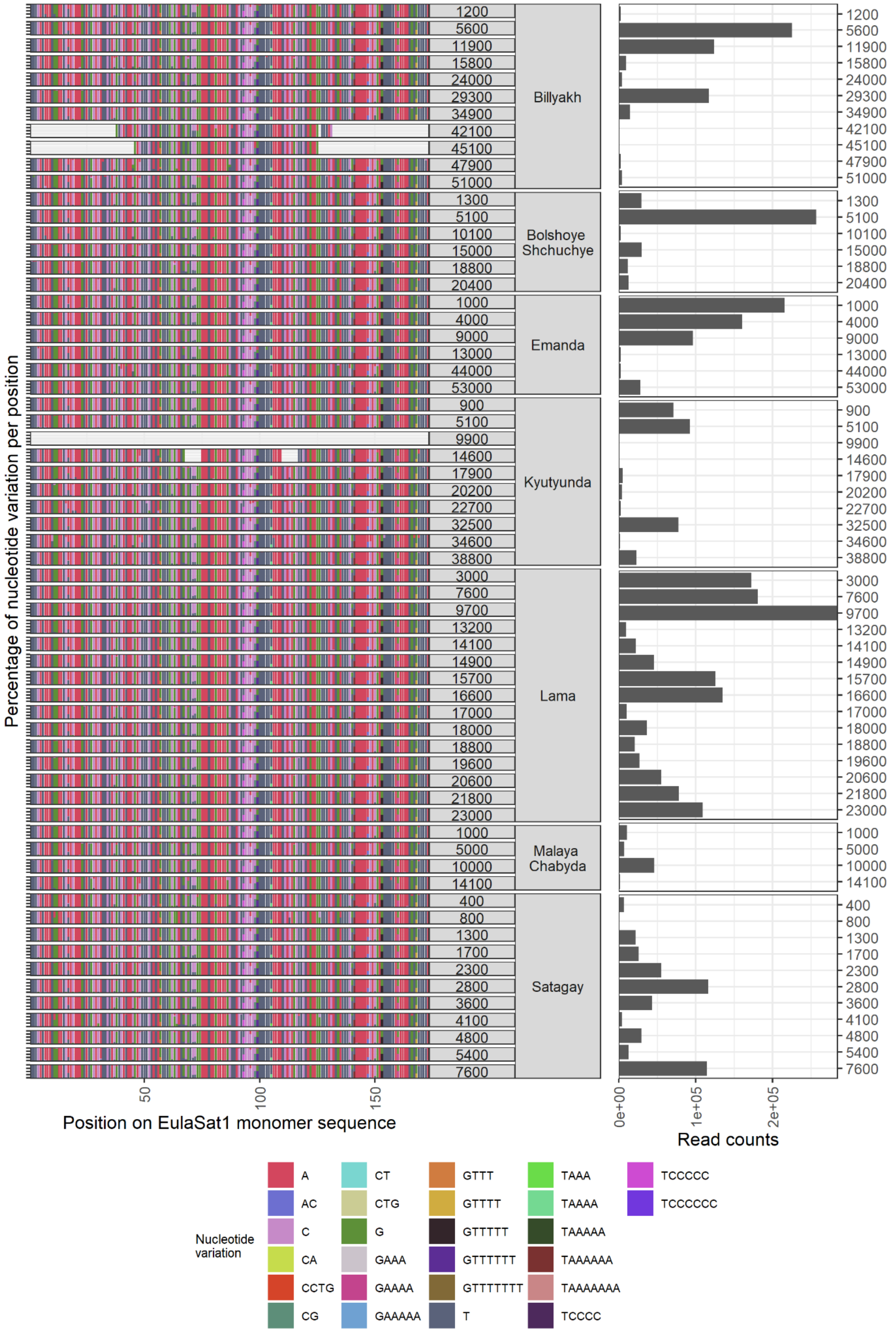

Percentage of nucleotide variation per position
Position on EulaSat1 monomer sequence
50
100
150
Billyakh
1200
5600
11900
15800
24000
29300
34900
42100
45100
47900
51000
Bolshoye Shchuchye
1300
5100
10100
15000
18800
20400
Emanda
1000
4000
9000
13000
44000
53000
Kyutyunda
900
5100
9900
14600
17900
20200
22700
32500
34600
38800
Lama
3000
7600
9700
13200
14100
14900
15700
16600
17000
18000
18800
19600
20600
21800
23000
Malaya Chabyda
1000
5000
10000
14100
Satagay
400
800
1300
1700
2300
2800
3600
4100
4800
5400
7600
Read counts
0e+00
1e+05
2e+05
Nucleotide variation
A
AC
C
CA
CCTG
CG
CT
CTG
G
GAAA
GAAAA
GAAAAA
GTTT
GTTTT
GTTTTT
GTTTTTT
GTTTTTTT
T
TAAA
TAAAA
TAAAAA
TAAAAAA
TAAAAAAA
TCCCC
TCCCCC
TCCCCCC

Fig. S4 Alignment of reads against the EulaSat1 repeat sequence. Left: Percentage of variations on the 173 positions of the repeat monomer arranged by calibrated age before present per lake, light gray background indicates positions with no coverage. Right: total read counts per sample.

Ancient damage patterns

Ancient damage patterns in the alignment of *Larix*-classified reads against the *Larix* chloroplast genome are visible for all samples with sufficient coverage (Supplementary Data 5). As typical for single-stranded library preparation methods, Cytosine (C) to Thymine (T) mutations are visible both at the 5' and 3' ends of the molecules[55]. Patterns are best visible in the biggest read fraction of overlapping merged reads. For high coverage samples, unmerged and unpaired reads also show similar patterns. When looking at samples within one lake, the C to T mutation rate often increases with age, but this trend is not consistent in all samples and especially not in low coverage samples.

Evaluation of negative controls

For each library batch of 7 samples, one library was built only with HPLC grade water (Sigma Aldrich, USA) instead of sample DNA. The library blanks have very few reads that map to the chloroplast genome (1-5 reads) but most of them (8 from in total 12) have no reads mapping. For each DNA extraction of 9 samples, one negative control without sediment was included. As samples from many different extraction batches were combined in this study, in some cases several extraction blanks were combined for one library preparation and therefore cannot be distinguished. In total 7 libraries with pools of extraction blanks were built. Two of the extraction blank libraries have no reads mapping to *Larix* (blanks for Lake Satagay, and one of the two blanks for Lake Billyakh). However, four of the extraction blank libraries have read counts mapping to the *Larix* chloroplast genome ranging from 49 to 94 and one of the libraries has 237 reads mapping to the reference (extraction blank for Lake Bolshoye Shchuchye). Considering that some of the low count samples have counts in the same range, a more detailed evaluation (see next section) is needed to determine whether any cross contamination occurred. Special attention is given to the signal of variation attributed to *L. sibirica,* which is one of the main results of the study.

Extraction batches showing potential cross contamination

For Lake Kyutyunda, samples were extracted in two extraction batches (LS130E and LS131E). In one batch (LS131E), the extraction blank library (JK102L-7) is extremely clean (the batch containing all the younger samples). The batch with the contaminated extraction blank library (JK106L-7-WDH) contains the older samples (samples of 38, 34 and 32 ka BP, extraction batch LS130E), which were extracted together with samples from Lake Malaya Chabyda. Samples of the two lakes show different signals, with Lake Kyutyunda exhibiting high proportions of *L. sibirica* variation and Lake Malaya Chabyda samples not showing any strong *L. sibirica* signal. Therefore, a cross-contamination between lakes is highly unlikely and the interpreted signal of *L. sibirica* in Lake Kyutyunda samples could not have come from a different source.

There are two libraries with pooled extraction blanks for Lake Billyakh. One of the blanks is extremely clean (JK102L-7, batch LS083E-LS131E-LS133E). This blank is an extraction blank for samples of ages 1, 5, 11 and 24 ka BP. The other blank is an extraction blank for samples of ages 34, 45, 47 and 51 ka BP (JK101L7-WDH, batch LS004E-LS005E-LS011E-LS022E). This means that the old samples are independent from the young samples. The samples from the blank in question were extracted in separate batches, each batch containing samples in a chronological manner. So, if there has been cross contamination, it is roughly from the same age/core depth and thus does not influence the interpretation of the observed patterns.

Samples of Lake Emanda were all extracted in one batch with one extraction blank. Cross contamination between blanks could have happened between the samples. However, none of the samples, nor the blank shows a strong signal of *L. sibirica* variation, so any potential cross contamination did not affect the interpretation of the results.

The blank for samples from Lake Satagay was extremely clean, cross-contamination is therefore highly unlikely.

The library of extraction blanks for Lake Bolshoye Shchuchye shows the highest read counts of the sequenced blanks. The blank also combines the highest number of extraction blanks pooled for one library. The whole core was extracted in a chronological manner, as a result all samples stem from different extraction batches. A possible cross-contamination in the course of the DNA extraction could have happened only between samples of similar age, and not between samples included in our study.

Exclusion of reads present in negative controls

To check whether the reads present in the extraction blank libraries had an impact on the results, we subtracted those reads from the sample reads. In detail, for each sample the extraction blank library containing the negative control of the respective extraction batch was selected. Each position covered in the extraction blank library was completely excluded from the belonging samples. The result is displayed in Fig. S5 and shows no difference in the interpreted results from the unfiltered samples.

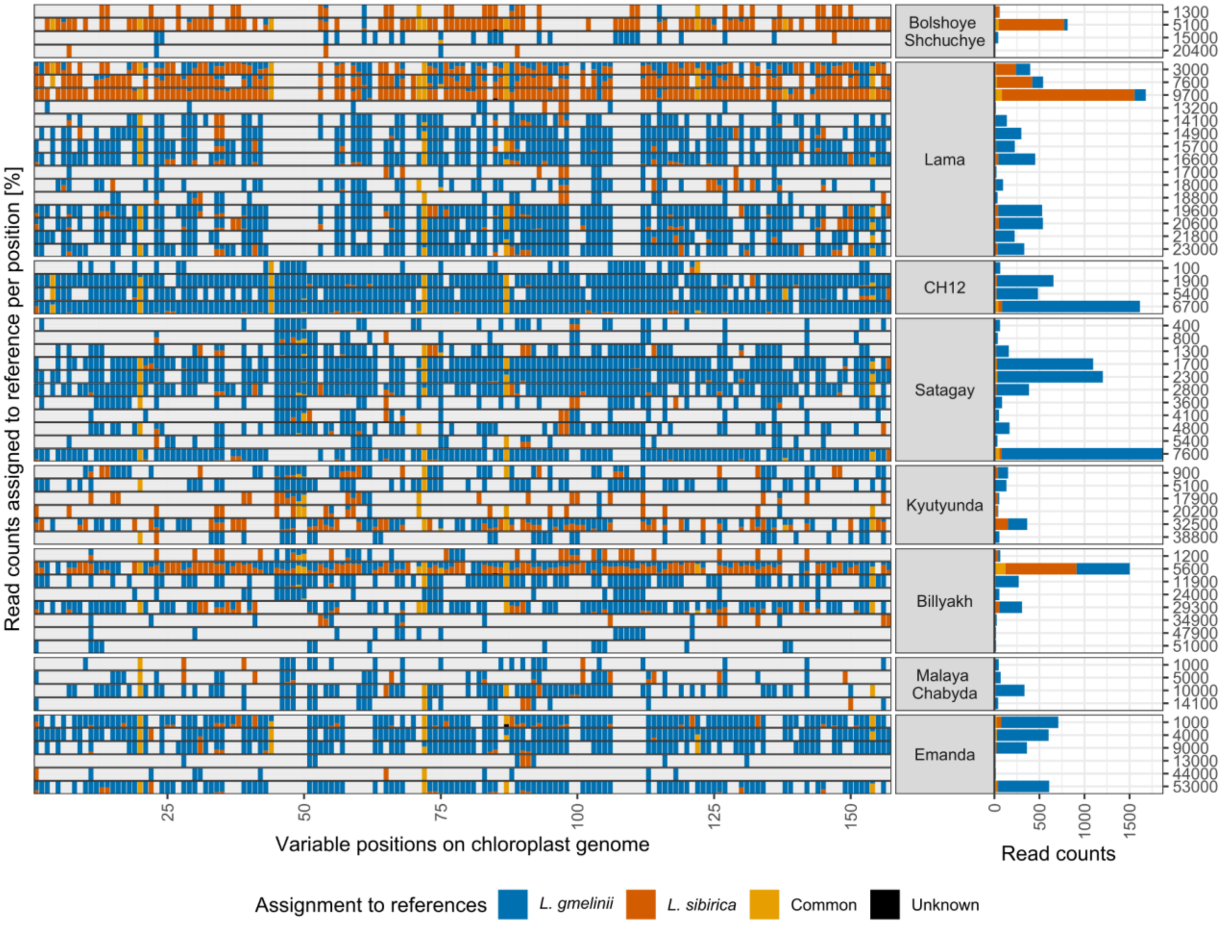

Fig. S5: Percentage and counts of variable positions on the chloroplast genome assigned to reference species with exclusion of blanks. Left: Alignment of *Larix*-classified reads against the chloroplast genome. Positions that are covered in the blank libraries are excluded from the corresponding samples. Only 157 variable positions between the reference species were considered. Colors indicate the percentage of variations at the positions assigned to the different *Larix* species. Each row represents one sample, arranged by calibrated age before present per lake. Gray background indicates positions with no coverage. Right: Number of read counts at the variable positions assigned to references for each sample.

Supplementary References

1. Blaauw, M. & Christen, J. A. Bacon. 1–15 (2011).
2. Andrews, S. FastQC: A quality control tool for high throughput sequence data. (2015).
3. Bushnell, B. BBMap. (2019).
4. Chen, S., Zhou, Y., Chen, Y. & Gu, J. Fastp: An ultra-fast all-in-one FASTQ preprocessor. *Bioinformatics* **34**, i884–i890 (2018).
5. Wood, D. E., Lu, J. & Langmead, B. Improved metagenomic analysis with Kraken 2. *Genome Biol.* **20**, 257 (2019).
6. O'Leary, N. A. *et al.* Reference sequence (RefSeq) database at NCBI: Current status, taxonomic expansion, and functional annotation. *Nucleic Acids Res.* **44**, D733–D745

(2016).
7. Lu, J. KrakenTools. (2020).
8. Li, H. & Durbin, R. Fast and accurate short read alignment with Burrows–Wheeler transform. *Bioinformatics* **25**, 1754–1760 (2009).
9. Oliva, A., Tobler, R., Cooper, A., Llamas, B. & Souilmi, Y. Systematic benchmark of ancient DNA read mapping. *Brief. Bioinform.* **00**, 1–12 (2021).
10. Li, H. *et al.* The Sequence Alignment/Map format and SAMtools. *Bioinformatics* **25**, 2078–2079 (2009).
11. Broad Institute. Picard toolkit. *GitHub-Repository* (2019).
12. Jónsson, H., Ginolhac, A., Schubert, M., Johnson, P. L. F. & Orlando, L. MapDamage2.0: Fast approximate Bayesian estimates of ancient DNA damage parameters. *Bioinformatics* **29**, 1682–1684 (2013).
13. Garrison, E. & Marth, G. Haplotype-based variant detection from short-read sequencing. 1–9 (2012).
14. Chang, C. C. *et al.* Second-generation PLINK: Rising to the challenge of larger and richer datasets. *Gigascience* **4**, 1–16 (2015).
15. Danecek, P. *et al.* The variant call format and VCFtools. *Bioinformatics* **27**, 2156–2158 (2011).
16. R Core Team. R: A language and environment for statistical computing. (2013).
17. Wickham, H. *et al.* Welcome to the Tidyverse. *J. Open Source Softw.* **4**, 1686 (2019).
18. Wickham, H. & Bryan, J. readxl: Read Excel Files. *R package version 1.3.1* https://cran.r-project.org/package=readxl (2019).
19. Wilke, C. O. cowplot: Streamlined Plot Theme and Plot Annotations for ‘ggplot2’. *R package version 1.1.1* https://cran.r-project.org/package=cowplot (2020).
20. van den Brand, T. ggh4x: Hacks for ‘ggplot2’. *R package version 0.1.2.1* https://cran.r-project.org/package=ggh4x (2021).
21. Okabe, M. & Ito, K. Color Universal Design (CUD) - How to make figures and presentations that are friendly to Colorblind people -. https://jfly.uni-koeln.de/color/ (2002).
22. Kahle, D. & Wickham, H. ggmap: Spatial Visualization with ggplot2. *R J.* **5**, 144–161 (2013).
23. Bivand, R., Keitt, T. & Rowlingson, B. rgdal: Bindings for the ‘Geospatial’ Data Abstraction Library. *R package version 1.5-23* https://cran.r-project.org/package=rgdal (2021).
24. Bivand, R., Pebesma, E. & Gomez-Rubio, V. *Applied spatial data analysis with R*. (Springer, 2013).
25. Robinson, D., Hayes, A. & Couch, S. broom: Convert Statistical Objects into Tidy Tibbles. *R package version 0.7.6* https://cran.r-project.org/package=broom (2021).
26. Yu, G. scatterpie: Scatter Pie Plot. *R package version 0.1.6* https://cran.r-project.org/package=scatterpie (2021).
27. Kearse, M. *et al.* Geneious Basic: An integrated and extendable desktop software platform for the organization and analysis of sequence data. *Bioinformatics* **28**, 1647–1649 (2012).
28. Hizume, M. *et al.* Tandem repeat DNA localizing on the proximal DAPI bands of chromosomes in \textit{Larix, Pinaceae}. *Genome* **45**, 777–783 (2002).
29. Heitkam, T. *et al.* Comparative Repeat Profiling of Two Closely Related Conifers (Larix decidua and Larix kaempferi) Reveals High Genome Similarity With Only Few Fast-Evolving Satellite DNAs. *Front. Genet.* **12**, 2021.03.21.436054 (2021).
30. Schulte, L. *et al.* Hybridization capture of larch (Larix Mill.) chloroplast genomes from sedimentary ancient DNA reveals past changes of Siberian forest. *Mol. Ecol. Resour.* **21**, 801–815 (2021).
31. Novák, P., Neumann, P., Pech, J., Steinhaisl, J. & MacAs, J. \textit{RepeatExplorer}: A

Galaxy-based web server for genome-wide characterization of eukaryotic repetitive elements from next-generation sequence reads. *Bioinformatics* **29**, 792–793 (2013).
32. Bolger, A. M., Lohse, M. & Usadel, B. Trimmomatic: A flexible trimmer for Illumina sequence data. *Bioinformatics* **30**, 2114–2120 (2014).
33. Langmead, B. & Salzberg, S. L. Fast gapped-read alignment with Bowtie 2. *Nat. Methods* **9**, 357–360 (2012).
34. Epp, L. S. *et al.* Temporal and spatial patterns of mitochondrial haplotype and species distributions in Siberian larches inferred from ancient environmental DNA and modeling. *Sci. Rep.* **8**, 17436 (2018).
35. Clarke, C. L. *et al.* Persistence of arctic-alpine flora during 24,000 years of environmental change in the Polar Urals. *Sci. Rep.* **9**, 19613 (2019).
36. Taberlet, P. *et al.* Power and limitations of the chloroplast trnL (UAA) intron for plant DNA barcoding. *Nucleic Acids Res.* **35**, e14–e14 (2007).
37. De Barba, M. *et al.* DNA metabarcoding multiplexing and validation of data accuracy for diet assessment: Application to omnivorous diet. *Mol. Ecol. Resour.* **14**, 306–323 (2014).
38. Binladen, J. *et al.* The use of coded PCR primers enables high-throughput sequencing of multiple homolog amplification products by 454 parallel sequencing. *PLoS One* **2**, 1–9 (2007).
39. Zimmermann, H. H. *et al.* Sedimentary ancient DNA and pollen reveal the composition of plant organic matter in Late Quaternary permafrost sediments of the Buor Khaya Peninsula (north-eastern Siberia). *Biogeosciences* **14**, 575–596 (2017).
40. Boyer, F. *et al.* OBITOOLS : a UNIX-inspired software package for DNA metabarcoding. *Mol. Ecol. Resour.* **16**, 176–182 (2016).
41. Sonstebo, J. H. *et al.* Using next-generation sequencing for molecular reconstruction of past Arctic vegetation and climate. *Mol. Ecol. Resour.* **10**, 1009–1018 (2010).
42. Willerslev, E. *et al.* Fifty thousand years of Arctic vegetation and megafaunal diet. *Nature* **506**, 47–51 (2014).
43. Soininen, E. M. *et al.* Highly Overlapping Winter Diet in Two Sympatric Lemming Species Revealed by DNA Metabarcoding. *PLoS One* **10**, e0115335 (2015).
44. Kanz, C. *et al.* The EMBL Nucleotide Sequence Database. *Nucleic Acids Res.* **33**, D29-33 (2005).
45. Biskaborn, B. K. *et al.* Late Quaternary vegetation and lake system dynamics in north-eastern Siberia: Implications for seasonal climate variability. *Quat. Sci. Rev.* **147**, 406–421 (2016).
46. Berglund, B. E. & Ralska-Jasiewiczowa, M. Pollen analysis and pollen diagrams. in *Handbook of Holocene palaeoecology and palaeohydrology.* (ed. Berglund, B. E.) 455–484 (Wiley, Chichester, 1987).
47. J., S. Tablets with spores used in absolute pollen analysis. *Pollen et Spores* **13**, 614–621 (1971).
48. Kupriyanova, L. A. & Alyoshina, L. A. *Pollen and spores of plants from the flora of European part of USSR*. (Academy of Sciences USSR, Komarov Botanical Institute, 1978).
49. Bobrov, A. E., Kupriyanova, L. A. & Litvintseva, M. V. *Spores and pollen of gymnosperms from the flora of the European part of the USSR*. (Nauka, 1983).
50. Reille, M. *Pollen et spores d'Europe et d'Afrique du nord. Supplement 2*. (Laboratoire de Botanique Historique et Palynologie, 1998).
51. Reille, M. *Pollen et spores d'Europe et d'Afrique du nord Supplement 1*. (Laboratoire de Botanique Historique et Palynologie, 1995).
52. Reille, M. *Pollen et spores d'Europe et d'Afrique du nord*. (Laboratoire de Botanique Historique et Palynologie, 1992).
53. Van Geel, B. Non-Pollen Palynomorphs. in *Tracking Environmental Change Using Lake*

Sediments. Developments in Paleoenvironmental Research (eds. Smol, J. P., Birks, H. J. B., Last, W. M., Bradley, R. S. & Alverson K.) 99–119 (Springer, Dortrecht, 2002). doi:10.1007/0-306-47668-1_6.
54. Grimm, E. C. TGView. (2004).
55. Gansauge, M.-T. & Meyer, M. Single-stranded DNA library preparation for the sequencing of ancient or damaged DNA. *Nat. Protoc.* **8**, 737–748 (2013).
