# Supplementary material for: Larix species range dynamics in Siberia since the Last Glacial captured from sedimentary ancient DNA
Source: Commun Biol. 2022 Jun 9;5:570. doi: 10.1038/s42003-022-03455-0 (PMC9184489; doi:10.1038/s42003-022-03455-0)
Supplement: Supplementary file 3 — Description of Additional Supplementary Files [file 42003_2022_3455_MOESM3_ESM.pdf]

Description of Additional Supplementary Files

File name: Supplementary Data 1
Description: Overview of sequencing results and classification and mapping using the chloroplast database and the chloroplast genome as reference.

File name: Supplementary Data 2
Description: Overview of sequencing results and classification and mapping using the extended plant database and the EulaSat1 satellite repeat sequence as reference.

File name: Supplementary Data 3
Description: Ancient damage patterns in alignment of Larix-assigned reads against the Larix chloroplast genome.

File name: Supplementary Data 4
Description: Lake sediment cores used in this study.

File name: Supplementary Data 5
Description: Radiocarbon dating of Lake Satagay sediment record (core ID PG2361).

File name: Supplementary Data 6
Description: List of samples included in the study with additional information on laboratory handling.

File name: Supplementary Data 7
Description: List of primers used for production of the nuclear gene bait set.

File name: Supplementary Data 8
Description: List of amplicons used in nuclear gene bait set.

File name: Supplementary Data 9
Description: Overview of pools used in hybridization capture experiment.

File name: Supplementary Data 10
Description: Sample list of metabarcoding approach.

File name: Supplementary Data 11
Description: Counts of mapped DNA sequences per sample and position against the L. gmelinii chloroplast genome (NCBI GenBank accession number MK468637) and the assignment to references.

File name: Supplementary Data 12
Description: Pollen percentages of Lake Kyutyunda (core ID PG2023).

File name: Supplementary Data 13
Description: Percentages of pollen, metabarcoding and counts of capture data of Fig. 2.
