# Supplementary material for: Larix species range dynamics in Siberia since the Last Glacial captured from sedimentary ancient DNA
Source: Commun Biol. 2022 Jun 9;5:570. doi: 10.1038/s42003-022-03455-0 (PMC9184489; doi:10.1038/s42003-022-03455-0)
Supplement: Supplementary file 5 — Reporting Summary [file 42003_2022_3455_MOESM5_ESM.pdf]

nature portfolio

Corresponding author(s): Ulrike Herzschuh, Luise Schulte

Last updated by author(s): Mar 25, 2022

Reporting Summary

Nature Portfolio wishes to improve the reproducibility of the work that we publish. This form provides structure for consistency and transparency in reporting. For further information on Nature Portfolio policies, see our Editorial Policies and the Editorial Policy Checklist.

Statistics

For all statistical analyses, confirm that the following items are present in the figure legend, table legend, main text, or Methods section.

n/a	Confirmed	
☐	☒	The exact sample size (n) for each experimental group/condition, given as a discrete number and unit of measurement
☐	☒	A statement on whether measurements were taken from distinct samples or whether the same sample was measured repeatedly
☒	☐	The statistical test(s) used AND whether they are one- or two-sided<br>*Only common tests should be described solely by name; describe more complex techniques in the Methods section.*
☒	☐	A description of all covariates tested
☒	☐	A description of any assumptions or corrections, such as tests of normality and adjustment for multiple comparisons
☒	☐	A full description of the statistical parameters including central tendency (e.g. means) or other basic estimates (e.g. regression coefficient) AND variation (e.g. standard deviation) or associated estimates of uncertainty (e.g. confidence intervals)
☒	☐	For null hypothesis testing, the test statistic (e.g. F, t, r) with confidence intervals, effect sizes, degrees of freedom and P value noted<br>*Give P values as exact values whenever suitable.*
☒	☐	For Bayesian analysis, information on the choice of priors and Markov chain Monte Carlo settings
☒	☐	For hierarchical and complex designs, identification of the appropriate level for tests and full reporting of outcomes
☒	☐	Estimates of effect sizes (e.g. Cohen's d, Pearson's r), indicating how they were calculated

Our web collection on statistics for biologists contains articles on many of the points above.

Software and code

Policy information about availability of computer code

Data collection	No software was used.
Data analysis	Software: FastQC (0.11.9), Clumpify (v.38.87), FastP (0.20.1), Kraken2 (2.1.1), Krakentools (0.1), BWA (0.7.17), samtools (1.11), Picard (2.24.1), mapDamage (2.2.1), freebayes (1.3.2), plink (1.90b4), vcftools (0.1.16), Geneious Prime (2021.2.2), repeatExplorer2 (2.3.7), trimmomatic (0.39), bowtie2 (2.4.2), TGView (1.7.16), obitools (3.0.0b38), , R (4.0.3), R-packages: ggplot (2 3.3.3), dplyr (1.0.6), readr (1.4.0), readxl (1.3.1), cowplot (1.1.1), ggh4x (0.1.2.1), rgdal (1.5-23), sp (1.4-5), broom (0.7.6), scatterpie (0.1.6), rbacon (2.5.0)<br>Detailed description of methods is given in the supplementary material.

For manuscripts utilizing custom algorithms or software that are central to the research but not yet described in published literature, software must be made available to editors and reviewers. We strongly encourage code deposition in a community repository (e.g. GitHub). See the Nature Portfolio guidelines for submitting code & software for further information.

Data

Policy information about availability of data

All manuscripts must include a data availability statement. This statement should provide the following information, where applicable:

- Accession codes, unique identifiers, or web links for publicly available datasets
- A description of any restrictions on data availability
- For clinical datasets or third party data, please ensure that the statement adheres to our policy

The Illumina sequence data of the hybridization capture dataset, targeting both the chloroplast and a set of nuclear genes of Larix on 64 samples and 19 negative

controls from seven lake sediment records and the hybridization capture dataset, targeting only the set of nuclear genes of Larix on 4 samples and 2 negative controls from Lake CH12 are submitted to the European Nucleotide Archive under the project number PRJEB47872.

Field-specific reporting

Please select the one below that is the best fit for your research. If you are not sure, read the appropriate sections before making your selection.

☐ Life sciences ☐ Behavioural & social sciences ☒ Ecological, evolutionary & environmental sciences

For a reference copy of the document with all sections, see nature.com/documents/nr-reporting-summary-flat.pdf

Ecological, evolutionary & environmental sciences study design

All studies must disclose on these points even when the disclosure is negative.

Study description	We sequenced target enriched lake sediment samples covering the las 50,000 years before present (BP) originating from lakes across Siberia and inferred past Larix species dynamics.
Research sample	We analysed 67 samples from 8 Siberian lakes. Lakes were selected according to availability lake records, the covered age (preferentially covering the Last Glacial Maximum) and according to known Larix occurrence (excluding lakes were no or little Larix is present).
Sampling strategy	Lake sediment records were sampled starting in 5000 years time resolution for records with an available age model prior to sampling. Other cores were sampled in higher resolution.
Data collection	Sedimentary ancient DNA was extracted at the dedicated ancient DNA laboratory at Alfred Wegener Institute, research unit Potsdam, and sequenced by Fasteris SA Sequencing Service (Geneva, Switzerland)
Timing and spatial scale	DNA data were collected from ancient sedimentary DNA samples from across Siberia, spanning the last 50,000 years.
Data exclusions	No data was excluded.
Reproducibility	This is a large scale study based on sample material which is difficult to retrieve. Thus there is no repeat attempted. However, trends were confirmed in the study by comparing with available proxies as metabarcoding data and pollen.
Randomization	Randomization is not relevant, samples were chosen based on specific criteria.
Blinding	Blinding is not relevant, as there was no presupposed hypothesis.

Did the study involve field work? ☒ Yes ☐ No

Field work, collection and transport

Field conditions	Field works were perfomed by different groups under different conditions. All sediment cores included in this study were already used in other published research which include a detailed description of field conditions.
Location	Lake records included in this study are: Bolshoye Shchuchye (67.53°N, 66.18°E), Lama (69.32°N, 90.12°E), Kytuyunda (69.38° N,123.38°E), Satagay (64.10°N, 122.15°E), Billyakh (65.17°N, 126.47°E), Malaya Chabyda (61.96°N, 129.41°E) and Emanda (65.17°N, 135.45°E).
Access & import/export	Lake sediment records were collected and exported by different research groups, in agreement with the rules of Russia.
Disturbance	The field work was done with minimal disturbance to the areas and the sediment deposits.

Reporting for specific materials, systems and methods

We require information from authors about some types of materials, experimental systems and methods used in many studies. Here, indicate whether each material, system or method listed is relevant to your study. If you are not sure if a list item applies to your research, read the appropriate section before selecting a response.

Materials & experimental systems

n/a	Involved in the study
☒	☐ Antibodies
☒	☐ Eukaryotic cell lines
☒	☐ Palaeontology and archaeology
☒	☐ Animals and other organisms
☒	☐ Human research participants
☒	☐ Clinical data
☒	☐ Dual use research of concern

Methods

n/a	Involved in the study
☒	☐ ChIP-seq
☒	☐ Flow cytometry
☒	☐ MRI-based neuroimaging
